# Examining the Prevalence and Risk Factors of School Bullying Perpetration Among Chinese Children and Adolescents

**DOI:** 10.3389/fpsyg.2022.720149

**Published:** 2022-03-14

**Authors:** Jia Xue, Ran Hu, Lei Chai, Ziqiang Han, Ivan Y. Sun

**Affiliations:** ^1^Factor-Inwentash Faculty of Social Work, University of Toronto, Toronto, ON, Canada; ^2^Faculty of Information, University of Toronto, Toronto, ON, Canada; ^3^Department of Sociology, University of Toronto, Toronto, ON, Canada; ^4^School of Political Science and Public Administration, Shandong University, Jinan, China; ^5^Department of Sociology and Criminal Justice, University of Delaware, Newark, DE, United States

**Keywords:** school bullying behavior, cyberbullying, children, family environment, parenting

## Abstract

**Background and Objectives:**

School bullying threatens the health of children and adolescents, such as mental health disorders, social deviant behaviors, suicidal behaviors, and coping difficulties. The present study aims to address (1) prevalence rates of both traditional and cyber school bullying perpetration, and (2) the associations between self-control, parental involvement, experiencing conflicts with parents, experiencing interparental conflict, and risk behaviors, and school bullying perpetration among Chinese children and adolescents.

**Method:**

This study used data from a national representative school bullying survey (*n* = 3,675) among children and adolescents from all grades (primary school 4th grade to high school 12th grade) in seven cities in China. Negative binomial regression was used to estimate the effects of these predictive factors on traditional and cyber school bullying perpetration, respectively. Seven control variables were included, such as gender, boarding school, family socioeconomic status, and parents’ education levels.

**Results:**

The sample comprised 52% female, 18% at boarding school, 70% of the participants’ academic performance was average or above. Approximately 17.3% of the participants reported participating in traditional school bullying against their peers, and 7.8% perpetrated cyberbullying behaviors. Also, after controlling sociodemographic characteristics and high self-control, parental involvement reduced the likelihood of traditional and cyberbullying perpetrating. Experiencing interparental conflict and risk behavior was significantly associated with increased perpetration of traditional and cyber school bullying. We found that having a conflict with parents was significantly associated with cyberbullying perpetration.

**Implications:**

Findings have implications for practice. Anti-bullying intervention programs targeting this population should consider these factors. For example, school administrators may develop school programs involving parents in the efforts and interventions workshops improving children and adolescents’ levels of self-control. Limitations are also discussed.

## Introduction

School bullying is an important social problem affecting children and adolescents in China (Chai et al., 2020a,b). Face-to-face bullying or traditional bullying is defined as “(a) repeated incidents amongst the same bullies and victims over time; (b) a physical, verbal, relational, or social attack or intimidation that is intended to cause harm, distress, or fear to victims; and (c) an imbalance of power between bullies and victims that more powerful adolescents dominate less powerful ones.” (Cho, 2019, p. 285; also see Olweus, 1978). Cyberbullying refers to bullying behaviors delivered in electronic contexts, such as e-mail, blogs, and text messages (Kowalski et al., 2014), and has emerged as a phenomenon in the field (Chai et al., 2020a,b). For example, Chan and Wong (2015) find that traditional bullying perpetration range from 2 to 68%. The rates of cyberbullying perpetration range from 3 to 60% in a sample of children and adolescents from Mainland China, Hong Kong, and Taiwan. Bullying behaviors have negative consequences, such as psychosomatic symptoms (e.g., headache, abdominal pain, and sleep problem) (Li et al., 2019), mental health outcomes (e.g., depression, anxiety, suicidal thoughts) (Gower and Borowsky, 2013; Benedict et al., 2015; Weng et al., 2017), and health behavior problems (e.g., alcohol, cigarettes) (Topper et al., 2011; Sangalang et al., 2016). The examination of potential risk factors of school bullying perpetration is helpful to prevent adverse health and well-being consequences for children and adolescents. However, empirical evidence of risk factors associated with school bullying perpetration is limited in China. The present study aims to address the research gap by examining the effects of several predicting factors on school bullying perpetration among a large sample of Chinese children and adolescents, including the impact of self-control behaviors, parental involvement, conflicts with parents, interparental conflict, and risk behaviors.

## Literature Review

### Self-Control Behaviors

A growing body of research has observed that self-control relates to bullying perpetration (Chui and Chan, 2015; Moon and Alarid, 2015). Self-control is an intrapersonal characteristic that influences bullying involvement (Hemphill et al., 2014). According to self-control theory, children and adolescents with lower levels of self-control are less likely to engage in socially desirable behaviors. Given certain circumstances, these individuals tend to be involved in risk and criminal behaviors because of their greater tendency to be impulsive, self-centered, and short-sighted (Gottfredson and Hirschi, 1990). In addition, these individuals are less likely to fear the potential adverse consequences of violent behaviors, which contributes to a greater chance of engaging in risk activities (Reisig and Pratt, 2011; Turanovic and Pratt, 2014). For example, Chui and Chan (2015) measure the levels of self-control in a sample of 365 adolescents in Macau, China, which is estimated based on risk-taking activities, self-centeredness, and volatile tempers. The study shows that youth with low self-control are more likely to report traditional bullying perpetration. Likewise, using the EU Kids Online II study, Vazsonyi et al. (2012) found that low self-control is associated with higher risks of cyberbullying perpetration. Similar patterns have also been observed in recent longitudinal analyses (Cho, 2018; Cho et al., 2019; Cho and Rustu, 2020).

### Parental Involvement

Parental involvement is an important factor influencing bullying behaviors (Shetgiri et al., 2012; Espelage, 2014). According to social control theory, individuals are more likely to develop risk behaviors if they lack social bonds (Hirschi, 1969). Social control theory has four components: attachment, commitment, involvement, and belief. Each component has an independent effect on risk behaviors, but the combined effect is expected to be the greatest (Hirschi, 1969). Given that parents are the primary agents that help children develop socialization, parents’ involvement significantly affects their children’s behaviors (Cho and Lee, 2018). Research has suggested that parents of bullying perpetrators tend not to be actively involved in their children’s lives.

In contrast, youth’s parents who are actively involved in children’s lives are less likely to become bullies (Espelage, 2014). Empirical studies have found evidence supporting this claim. For instance, using data from the National Survey of Children’s Health, Shetgiri et al. (2012) found that parental involvement was associated with a lower likelihood of traditional bullying perpetration. Likewise, using two parent-child dyads studies, Barlett and Fennel (2018) showed that lack of parental involvement is linked to greater risks of cyberbullying perpetration. Similar patterns have also been observed in other studies (Cho et al., 2019; Paez, 2020).

### Conflict With Parents

The conflict between parents and their kids is another important factor contributing to the youth’s involvement in bullying. General strain theory posits that strains are essential factors resulting in risk behaviors; individuals who experience stresses tend to engage in risk activities as a coping strategy to respond to injustice (Agnew, 2002, 2006). Some studies have indicated that criminally victimized, or racially discriminated individuals are associated with a higher likelihood of committing deviant behaviors (Agnew et al., 2002; Baron, 2004; Moon et al., 2009). Within the context of bullying perpetration, researchers have identified that conflict with parents is a common strain factor shaping bullying behaviors among children and adolescents (Moon et al., 2012). Using the Korea Youth Survey, Moon et al. (2012) found that children who experienced conflict with parents were more likely to engage with traditional bullying perpetration. Likewise, a recent review piece demonstrates an inverse association between relationships with parents and cyberbullying perpetration (Camerini et al., 2020). Other studies have also observed a similar pattern (Stevens et al., 2000; Pepler et al., 2008).

### Interparental Conflict

Interparental conflict is an external family factor that impacts the youth’s bullying involvement (Gini et al., 2014). Interparental conflict refers to “verbal or physical assaults and disputes between parents due to disagreement or other reasons” (Yang et al., 2018, p. 257; also see Fincham, 1994). According to social learning theory, children can learn behaviors by observing their parents (Akers, 2009). They can apply what they have learned at home to their peers at school or online (Tanrikulu and Campbell, 2015). Research reveals that children who grow up in an interparental conflict environment are more likely to involve in bullying perpetration (Baldry, 2003; Low and Espelage, 2013; Yang et al., 2018; Hsieh et al., 2021). For instance, using a sample of elementary school children in South Korea, Shin et al. (2014) demonstrate that interparental conflict is linked to higher likelihoods of traditional bullying perpetration. Likewise, using a sample of 649 Chinese high school students, Yang et al. (2018) observed that interparental conflict is positively associated with cyberbullying perpetration among adolescents.

### Risk Behaviors

To reiterate, social learning theory posits that children tend to learn their behaviors based on observation (Bandura, 1978). In addition to observing the parents that shape children’s and adolescents’ behaviors are through social media platforms. Prior research has stressed that children and adolescents might engage in aggressive behaviors through media use (Bandura, 1978). More specifically, playing online games provide opportunities for youth to learn and develop aggression (Huesmann, 2007; Anderson et al., 2010; Lam et al., 2013). Chang et al. (2015) sampled 2,315 students in Taiwan and observed that online game use is associated with a higher likelihood of involving in cyberbullying perpetration. Lam et al. (2013) found that youth exposed to violent online games are more likely to engage in cyberbullying perpetration. A recent piece also finds a similar pattern (Teng et al., 2020). Although existing literature has documented the association between online games and cyberbullying perpetration, some researchers have proposed that the adverse effect of online games might apply to real-time (Boyd and Swanson, 2016). In addition, a growing body of research links substance use (e.g., drinking and smoking) to various health-risk behavioral outcomes among youth (Ellickson et al., 2003; Cho et al., 2007; Swahn et al., 2008). However, little is known about whether the same detrimental patterns can be applied to another important but as yet understudied health-risk behavior—bullying perpetration. Some preliminary evidence is that youth’s alcohol use was positively associated with bullying perpetration (Swahn et al., 2011).

### Aim of the Study

The present study proposes five hypotheses based on existing empirical research as follows:

Hypothesis 1: Low self-control is more likely to associate with both traditional bullying and cyberbullying perpetration.
Hypothesis 2: Parental Involvement is associated with a lower likelihood of both traditional bullying and cyberbullying perpetration.
Hypothesis 3: Experiencing conflict with parents is more likely to perpetrate traditional bullying and cyberbullying.
Hypothesis 4: Experiencing interparental conflict is positively associated with traditional bullying and cyberbullying perpetration.
Hypothesis 5: Risk Behaviors are positively associated with both traditional bullying and cyberbullying perpetration.

## Methods

### Sample

The present study used data drawn from a nationwide research project conducted in 2016 in seven regions of China, including the capital city (Beijing) and six provinces (Liaoning, Hunan, Jiangsu, Guangdong, Guizhou, and Gansu^1^). For geographical variety, we purposively selected them using stratified sampling. In each of the seven cities, we selected a primary school, a middle school, a high school, and a vocational school^2^. Then, we randomly chose one class for each grade from these sampled schools. Then we invited the students from the sampled classes to complete the survey. Grades one to three students were not a part of the sample because they could not read and understand the survey questions. The sampling strategy was chosen to best balance the “representativeness,” the scientific rationale, and the available reality (Lohr, 2009).

Research assistants distributed paper questionnaires to students at each school site. We informed the participants that participation in the study was voluntary. In addition, we also obtained consent from their parents and teachers. Students who consented to participate in the study completed the questionnaire independently. The research team collected a total of 3,777 questionnaires across all school sites, of which 3,675 were valid. Therefore, the sample comprised 3,675 students in the present study. Slightly over half of the sample were female (52%, *n* = 1,903) and 48% (*n* = 1,772) were male students. For educational level, 38% (*n* = 1,388) were attending primary school at the time of the survey, 28% were in middle school (*n* = 1,020), and 34% were in either high school (*n* = 1,089) or vocational training school (*n* = 178). Table 1 described the sociodemographic characteristics of the sample.

**TABLE 1:** Descriptive statistics of variables (*N* = 3,675).

Variables	Mean	SD	Range	Cronbach’s Alpha
***Dependent variables***				
Traditional bullying	0.40	1.11	0–6	0.88
Cyberbullying	0.17	0.68	0–4	0.91
***Independent variables***				
Parental attachment	19.00	3.92	6–24	0.85
Conflict with parents	0.21	0.41	0–1	–
Inter-parental conflict	0.17	0.38	0–1	–
Self-control	21.17	4.83	7–28	0.81
Risk behaviors	9.04	2.96	7–28	0.72
***Control variables***				
	***n***	**%**		
Gender				
Male	1,764	48		
Female	1,911	52		
**Boarding at school**				
Yes	662	18		
No	3,013	82		
**Grade level**				
Elementary school*	1,388	37.77	–	–
Middle school	1,020	27.76	–	–
High school	1,267	34.48	–	–
**Academic performance**				
Top of the class*	361	9.82	–	–
Above average	1,153	31.37	–	–
Average	1,416	38.53	–	–
Below average	542	14.75	–	–
Bottom of the class	203	5.52	–	–
**Father’s education level**				
Below middle school*	370	10.07	–	–
Middle school	1,152	31.35	–	–
High/vocational school	891	24.24	–	–
College	935	25.44	–	–
Graduate or above	327	8.90	–	–
**Mother’s education level**				
Below middle school*	527	14.34	–	–
Middle school	1,103	30.01	–	–
High/vocational school	819	22.29	–	–
College	919	25.01	–	–
Graduate or above	307	8.35	–	–
**Family economic status**				
Poor or below average*	545	14.83	–	–
Average	1,992	54.20	–	–
Above average	970	26.39	–	–
Very well	168	4.57	–	–

*Reference group in regression analysis.

### Measures

#### Dependent Variables

Two dependent variables examined in the study were (a) traditional bullying behavior and (b) cyberbullying behavior. Participants were invited to recall their bullying behavior that had occurred in the past year at the time of the survey. Questions on both scales were adapted from the National Center for education Statistics’ School Survey on Crime and Safety (Robers et al., 2014). Participants were asked, “In this past year, have you done any of the following to any of your classmates?” A frequency Likert scale was used for each item (0 = *never*, 1 = *occasionally*, 2 = *sometimes*, 3 = *frequently*). For the present study, we created two count outcome variables with each item was dichotomized first and then summed up. Traditional bullying behavior (α = 0.88) included six items representing six different types of bullying behavior: (1) making fun of other students in a hurtful way, (2) spreading rumors about other students, (3) threatening others, (4) physically pushing, shoving, striping, or spitting on others, (5) isolating others on purpose, and (6) damaging others’ belongings. The six items were summed up to construct the dependent variable, traditional bullying behavior. Cyberbullying behavior included four types (α = 0.91): (1) making fun of other students online, (2) threatening or insulting online, (3) spreading rumors or disclosing private information about others online, and (4) isolating other students online. The four items were summed up to construct the dependent variable, cyberbullying behavior.

#### Independent Variables

There were five independent variables, including self-control, parental involvement, experiencing conflict with parents, experiencing interparental conflict, and risk behaviors.

We measured self-control by summing up seven items (α = 0.85): (1) I am an impulsive person; (2) when tasks get challenging or complicated, I tend to give up; (3) When I am angry at people, I feel more like hurting them than talking to them about why I am upset; (4) I lose my temper easily; (5) it is hard for me to have empathy for people in difficult situations; (6) when I am angry, people better stay away from me; and (7) when I disagree with others, I often do not give in.

Parental involvement was constructed by summing up six items (α = 0.85): (1) my parents know most of my friends, (2) my parents usually know where I go if I am not home, (3) my parents spend a lot of time with me, (4) my parents chat with me often, (5) my parents encourage me often, and (6) I feel my parents care for me. Each item was assessed using a Likert scale from 1 to 4 (1 = *strongly disagree*, and 4 = *strongly agree*).

The team created one item to measure participants’ experience conflict with their parents, “I have a conflict with my parents often.” This item is a binary (1 = *yes*, 0 = *no*).

We also created a single binary item to measure experiencing the interparental conflict, “my parents fight often” (1 = *yes*, 0 = *no*).

Risk behavior was constructed by summing up six items (α = 0.76): (1) truancy, (2) smoking, (3) street fight, (4) alcohol use, (5) excessive online gaming, and (6) did not fasten the safety belt while sitting in the front seat. A frequency Likert scale was used for each item (1 = *absolutely agree*, 2 = *agree*, 2 = *disagree*, 3 = *absolutely disagree*).

#### Control Variables

There were 7 control variables, including gender, schooling, boarding at school, father’s education, mother’s education, self-rated family economic status and academic performance. Gender was a binary variable (0 = *female*, 1 = *male*). Schooling included three categories^3^ (1 = *primary school*, 2 = *middle school*, and 3 = *high school*). Boarding at school is a binary variable (1 = *yes*, 0 = *no*). Father’s education included five levels (1 = *below middle school*, 2 = *completed middle school*, 3 = *completed high/vocational training school*, 4 = *completed college*, and 5 = *completed graduate school or above*). Mother’s education included five categories (1 = *below middle school*, 2 = *completed middle school*, 3 = *completed high/vocational training school*, 4 = *completed college*, and 5 = *completed graduate school or above*). Family economic status were self-reported, including four levels (1 = *poor or way below average*, 2 = *average*, 3 = *above average*, and 4 = *very well*). Academic performance was a self-rated five-level variable (1 = *top of the class*, 2 = *above average*, 3 = *average*, 4 = *below average*, and 5 = *bottom of the class*). We presented the results in Table 1.

### Analytical Plan

Negative binomial regression models with robust error variance were used to examine the effects of parental involvement, conflict with parents, risk behavior, self-control, interparental conflict, and conflict with parents on traditional school bullying and cyberbullying, respectively. Negative binomial models were selected over Poisson regression models for the two count outcome variables because (1) incidents of bullying perpetration remained low among students, resulting in the skewness of the distribution, and (2) the issue of overdispersion (Huang and Cornell, 2012). All analyses were conducted using Stata 16.1.

## Results

### Traditional Bullying Perpetration

We found that 17.3% of the respondents reported having participated in any traditional forms of bullying behavior. Parental involvement reduced the likelihood of traditional bullying behavior (*b* = −0.108, *SE* = 0.012, *p* = 0.000, AME = −0.063) after controlling sociodemographic variables. Self-control was also negatively associated with traditional bullying behavior (*b* = −0.095, *SE* = 0.01, *p* = 0.000, AME = −0.055). Those who reported witnessing interparental conflict at home were reporting more traditional bullying perpetration (*b* = 0.34, *SE* = 0.113, *p* = 0.003, AME = 0.217). The association between experiencing conflicts with parents and traditional bullying perpetration was marginally significant (*b* = 0.207, *SE* = 0.109, *p* = 0.058, AME = 0.126). Risk behavior was also positively associated with increased traditional bullying perpetration (*b* = 0.215, *SE* = 0.02, *p* = 0.000, AME = 0.126).

### Cyberbullying Perpetration

When examining the prevalence rates of children and adolescents’ participation in bullying behavior, 7.8% of the respondents reported that they have participated in cyberbullying in the past year. Table 2 presented the results of the two negative binomial regression models.

**TABLE 2:** Negative binomial regression models predicting traditional bullying and cyberbullying (*N* = 3,675).

	Traditional bullying				Cyberbullying			
	*B (SE)*	*p*	95% CIs	AME	*B*	*p*	95% CIs	AME
***Independent variables***								
Parental involvement	−0.108*** (0.012)	0.000	[−0.132, −0.084]	−0.063	−0.118***(0.02)	0.000	[−0.157, −0.079]	−0.013
Conflicts with parents	0.207 (0.109)	0.058	[−0.007, 0.421]	0.126	0.331*(0.163)	0.042	[0.011, 0.65]	0.09
Interparental conflict	0.34**(0.113)	0.003	[0.118, 0.562]	0.217	0.382*(0.185)	0.039	[0.02, 0.744]	0.108
Self-control	−0.095***(0.01)	0.000	[−0.114, −0.075]	−0.055	−0.126***(0.015)	0.000	[−0.155, −0.097]	−0.033
Risk behaviors	0.215***(0.02)	0.000	[0.176, 0.253]	0.126	0.232***(0.027)	0.000	[0.179, 0.286]	0.061
**Control variables**								
Male	0.209*(0.099)	0.034	[0.016, 0.403]	0.119	0.453**(0.152)	0.003	[0.154, 0.751]	0.108
Boarding at school	0.398** (0.136)	0.003	[0.132, 0.665]	0.264	0.229 (0.191)	0.232	[−0.146, 0.604]	0.064
**Grade level**								
Middle school	−0.98***(0.125)	0.000	[−1.226, −0.734]	−0.686	−0.472*(0.195)	0.016	[−0.855, −0.089]	−0.139
High school	−1.486***(0.123)	0.000	[−1.728, −1.244]	−0.850	−0.686***(0.18)	0.000	[−1.038, −0.334]	−0.183
**Academic performance**								
Above average	0.109 (0.19)	0.566	[−0.264, 0.482]	0.063	−0.089 (0.262)	0.734	[−0.602, 0.424]	−0.025
Average	0.044 (0.187)	0.813	[−0.322, 0.41]	0.025	−0.19 (0.264)	0.471	[−0.706, 0.327]	−0.050
Below average	0.075 (0.209)	0.721	[−0.336, 0.485]	0.042	−0.048 (0.298)	0.872	[−0.631, 0.535]	−0.014
Bottom of the class	0.122 (0.24)	0.612	[−0.349, 0.593]	0.071	−0.169 (0.37)	0.648	[−0.893, 0.556]	−0.045
**Father’s education level**								
Middle school	−0.486** (0.156)	0.002	[−0.793, −0.18]	−0.331	−0.344 (0.242)	0.154	[−0.819, 0.13]	−0.094
High/vocational school	−0.681*** (0.182)	0.000	[−1.037, −0.324]	−0.424	−0.48 (0.26)	0.065	[−0.99, 0.03]	−0.123
College	−0.4* (0.201)	0.047	[−0.794, −0.006]	−0.283	−0.117 (0.295)	0.693	[−0.694, 0.461]	−0.035
Graduate or above	−0.613* (0.284)	0.031	[−1.168, −0.057]	−0.393	0.198 (0.486)	0.684	[−0.754, 1.15]	0.071
**Mother’s education level**								
Middle school	−0.003 (0.153)	0.984	[−0.303, 0.297]	−0.002	0.017 (0.24)	0.943	[−0.453, 0.487]	0.005
High/vocational school	−0.126 (0.179)	0.482	[−0.476, 0.225]	−0.075	−0.145 (0.277)	0.602	[−0.687, 0.398]	−0.040
College	−0.281 (0.189)	0.138	[−0.652, 0.09]	−0.157	−0.256 (0.294)	0.384	[−0.833, 0.321]	−0.068
Graduate or above	−0.143 (0.294)	0.626	[−0.718, 0.432]	−0.085	−0.47 (0.482)	0.329	[−1.414, 0.474]	−0.112
**Family economic status**								
Average	−0.163 (0.132)	0.214	[−0.421, 0.094]	−0.100	−0.338 (0.201)	0.092	[−0.731, 0.055]	−0.094
Above average	−0.11 (0.151)	0.466	[−0.405, 0.185]	−0.069	−0.099 (0.225)	0.659	[−0.54, 0.342]	−0.031
Very well	−0.237 (0.261)	0.363	[−0.748, 0.274]	−0.140	−0.64 (0.399)	0.109	[−1.423, 0.143]	−0.156
**Likelihood-ratio test of overdispersion**	*G*^2^ = 639.70, *p* < 0.000					*G*^2^ = 1012.03, *p* < 0.000		

*p < 0.05, **p < 0.01, ***p < 0.001. AME, Average marginal effects. G^2^ = 2(ln⁡L_NBRM_−ln⁡L_NBR_).

After controlling sociodemographic characteristics, parental involvement was negatively associated with cyberbullying behavior (*b* = −0.118, *SE* = 0.02, *p* = 0.000, AME = −0.013). Self-control were negatively associated with cyberbullying behavior (*b* = −0.126, *SE* = 0.05, *p* = 0.000, AME = −0.033). Witnessing interparental conflict was positively associated with cyberbullying perpetration (*b* = 0.382, *SE* = 0.185, *p* = 0.039, AME = 0.108). Those who reported having conflicts with parents were also reporting increased cyberbullying perpetration (*b* = 0.331, *SE* = 0.163, *p* = 0.042, AME = 0.09). Last, results showed that risk behavior was also positively associated with increased traditional and cyberbullying behavior (*b* = 0.232, *SE* = 0.027, *p* = 0.000, AME = 0.061).

## Discussion

The present study contributes to the school bullying literature in China in two ways: (1) assessing the prevalence of both traditional and cyberbullying perpetration, and (2) examining risk factors contributing to bullying behaviors among Chinese children and adolescents using a national survey from China in 2016. About seventeen percent of the respondents reported having participated in any traditional forms of bullying behavior, and 7.8% of the respondents reported that they have participated in cyberbullying in the past year. Findings support our five hypotheses and are consistent with previous studies.

Findings support our hypothesis 1 that low self-control is more likely to associate with both traditional bullying and cyberbullying perpetration. Self-control has long been recognized as an important factor related to deviant behaviors (Gottfredson and Hirschi, 1990; Pratt and Cullen, 2000), such as school bullying behaviors in the present study (Chui and Chan, 2015; Moon and Alarid, 2015). Consistent with past findings, we find that participants with lower self-control scores are more likely to perpetrate both traditional and cyberbullying against their peers. Empirical evidence has supported that individuals with adequate self-control are likely to conform to social rules, and early efforts can be made toward correcting norm violations (Vazsonyi and Huang, 2010). In addition, a study in 25 European countries found no significant differences between females and males on the association between low self-control and cyberbullying perpetration (Vazsonyi et al., 2012). Future research may consider examining the gender differences in the links between self-control and school-based bullying among the Chinese population.

Findings support our hypotheses 2, 3, and 4 that Parental Involvement is associated with a lower likelihood of both traditional bullying and cyberbullying perpetration. Experiencing conflict with parents or interparental conflict is more likely to perpetrate traditional bullying and cyberbullying. These three factors focus on children’s and adolescents’ experiences of interactions with family, teachers, peers, or school environments in the field of school bullying research (Lee, 2011). We assessed three predictive factors in the present study: parental involvement, experiencing interparental conflict, and having a conflict with parents. The present study shows that parents’ involvement reduces the likelihood of both traditional and cyberbullying perpetration among children and adolescents. Parental involvement in this study refers to the social interconnections between more than one microsystem relationship (Lee, 2011). It is also worthy to note that some earlier studies operationalize parental involvement as parents communicate with teachers and peers at schools and observe that a lack of parent involvement is associated with increased bullying behaviors (Flouri and Buchanan, 2003). In contrast, the present study operationalizes parent involvement as a friendly familial interaction between parents and the kids, such as “parents and kids spend a lot of time together,” “parents chat often with kids,” or “kids feel their parents care for them.”

Respondents who reported having conflicts with parents also reported increased cyberbullying perpetration instead of traditional bullying perpetration. This finding conflicts with Moon et al. (2012), which finds Korean children experiencing conflict with parents are more likely to engage with traditional bullying perpetration. Our findings are consistent with other studies which reveal a positive association between having conflicts with parents and cyberbullying perpetration (Stevens et al., 2000; Pepler et al., 2008; Camerini et al., 2020).

Likewise, experiencing interparental conflict is associated with increased perpetration behaviors. Relevant studies have suggested that childhood exposure to domestic violence, having a poor relationship with parents, or lack of parental monitoring are more likely to bully their peers (Cho et al., 2019). Our findings confirm the significant role of a number of factors in shaping school bullying behaviors among children and adolescents throughout psychological growth (Bronfenbrenner, 1979).

Findings support our hypothesis 5 that risk behaviors are positively associated with traditional bullying and cyberbullying perpetration. Our designed risk behaviors items are adopted and revised based on CDC’s Youth Risk Behavior Survey (Brener et al., 2013). It is worth mentioning that excessive online gaming provides opportunities for children and adolescents to participate in risk behaviors via media use, contributing to the likelihood of increased cyberbullying behaviors. Our findings are consistent with existing studies among the Chinese population (Lam et al., 2013; Chang et al., 2015; Teng et al., 2020). Future research might explore further the real-time effects of online games on cyberbullying behaviors.

### Implications

This study has significant implications for practice and research. First, supportive familial relations and environment (e.g., minimizing exposure to domestic violence) and adequate parental supervision have been recognized as protective factors for bullying perpetration. Teachers and staff alone will not fundamentally mitigate school-based face-to-face or cyberbullying behaviors. School programs involving parents in efforts should be developed. For example, school administrators may consider inviting parents to their safety and health committee and raising parents’ awareness about the identified protective factors in empirical evidence in Chinese society. Besides, self-control can be improved through training programs or workshops at school. For example, anger management is suggested to be incorporated into these workshops because proper anger management intervention was reported in previous studies associated with a 60 to 70% decrease in the odds of physical assault (Xue et al., 2019).

Second, the present study does not examine the effects of any interaction terms on bullying. For example, self-control may interact with being at boarding school because the living arrangements by the school potentially affect students’ level of self-control (Chui and Chan, 2015). Besides, we find low self-control and having risk behaviors significantly contribute to participants’ bullying perpetration. Gottfredson and Hirschi (1990) claim that low self-control correlates with all sorts of deviant behaviors, such as truancy, street fight, or excessive online gaming in the present study. We hypothesize that low self-control affects these moderate risk behaviors, which would, in turn, influence school-based bullying perpetration. Future research may assess further the mediating effect of these proposed risk behaviors on self-control and bullying perpetration.

Last, limited research exists with regard to the examination of different levels of factors from the ecological model in school-based bullying. Since it is not the present study’s focus, we do not examine the effects of all levels of factors in the ecological system theory in school-based bullying behaviors. Future studies may consider employing this framework to assess bullying victimization and perpetration among Chinese children and adolescents. In particular, the interactions across the different levels of systems (Espelage, 2014).

### Limitations

There are several limitations in the study. First, the present study has a limitation of potential causality due to its cross-sectional nature. Second, participants’ self-reporting of conflict with parents, socio-economic status, and academic performance is based on their subjective perceptions, which may not fully reflect the actual situations. Last, we do not assess the victimization experience of the participants. Results may be interpreted differently when we consider the occurrence of both perpetration and victimization.

## Conclusion

The present study provides insights into understanding the school bullying perpetration among children and adolescents in China. More specifically, findings show that parental involvement and stronger self-control are critical to reducing traditional and cyberbullying perpetration. Besides, we find that participants who have conflicts with parents, witness interparental conflict, and experience risk behaviors positively predict increased traditional and bullying perpetration. Future studies should explore and develop the intervention school programs targeting this population.

## Data Availability Statement

The data analyzed in this study is subject to the following licenses/restrictions: Available upon request to protect participant’s privacy. Requests to access these datasets should be directed to ZH, ziqiang.han@sdu.edu.cn.

## Ethics Statement

The studies involving human participants were reviewed and approved by the Shandong University. Written informed consent to participate in this study was provided by the participants’ legal guardian/next of kin.

## Author Contributions

JX designed the study and wrote the manuscript. RH analyzed the data. LC wrote a portion of the manuscript. ZH and IS revised the manuscript. All authors contributed to the article and approved the submitted version.

## Conflict of Interest

The authors declare that the research was conducted in the absence of any commercial or financial relationships that could be construed as a potential conflict of interest.

## Publisher’s Note

All claims expressed in this article are solely those of the authors and do not necessarily represent those of their affiliated organizations, or those of the publisher, the editors and the reviewers. Any product that may be evaluated in this article, or claim that may be made by its manufacturer, is not guaranteed or endorsed by the publisher.

## Footnotes

1 Beijing is located in North China, and Liaoning, Hunan, Jiangsu, Guangdong, Guizhou, and Gansu are located in Northeast, South Central, eastern-central coastal, South, Southwest, and North-central China, respectively.

2 Vocational training schools in China are equivalent to high schools but are preparing students for specific vocational tracks rather than focusing on academics.

3 We included primary students from 4th grade to 6th grade. In China, primary school students from 4th to 6th grade are typically in the age range 9 – 12; middle school students are in the age range 13 – 15; high school students are in the age range 16 – 18.
